# Double talon cusps on supernumerary tooth fused to maxillary central incisor: Review of literature and report of case

**DOI:** 10.4317/jced.51428

**Published:** 2014-10-01

**Authors:** Faiez N. Hattab

**Affiliations:** 1 BDS, PhD, Odont. Dr., Professor and Senior Consultant in Restorative and Pediatric Dentistry, private practice, Amman, Jordan

## Abstract

Human tooth development is a continuous process begin at the sixth weeks in utero and extends to about sixth months after birth for the primary dentition and from sixteenth week in utero to late adolescence for permanent dentition. There is no other organ of the human body which takes so long to attain its ultimate morphology as dentition. Several physiologic growth processes participate in the progressive development of the teeth including: initiation, proliferation, histodifferentiation, morphodifferentiation, apposition, calcification, and eruption. Aberrations in different stages of tooth development can result in unique manifestations both in primary and permanent dentitions. The fact that premaxilla is the predilection site for the occurrence of supernumerary teeth, talon cusp, dens invaginatus, and geminated teeth may suggest that the embryological development of premaxilla differ from other sites of the jaws. The dental abnormalities presented in this review are of great concern to dentist and parents because they create clinical, pathological and esthetic problems. Dental practitioner should be aware of the clinical sign, associated problems and treatment options for a given case.

** Key words:**Double talon cusps, fusion, supernumerary, case report.

## Supernumerary Teeth (hyperdontia)

Supernumerary teeth are a developmental abnormality referred to teeth formed in excess number of that found in the normal dental formula. They are considered to be one of the most significant dental anomalies affecting the primary and mixed dentition because of the clinical problems they can create. Most problems associated with supernumerary teeth are due to their interference with the normal eruption and positioning of the adjacent teeth causing malocclusion. Ninety to 98% of all supernumeraries occur in the maxilla with strong predilection for the premaxillary region ranged between 65 and 90% (1-4). Supernumerary teeth may be single or multiple, unilateral or bilateral, malformed or normal in size and shape, erupted or impacted, and occurs in the maxilla, the mandible, or both. Single supernumerary occur in 64-86%, double supernumeraries in 12-23%, and multiple supernumeraries in 1-5% of the cases (4-6). Supernumerary teeth may erupt normally, remain impacted, appear inverted, or assume an abnormal path of eruption. In permanent dentition, only 13% of tuberculate supernumeraries, 28% of conical type and 62% of supplemental type are erupted ( Table 1), compared with 73% of primary supernumeraries mainly supplemental type (7). Delayed eruption of permanent incisors because of the presence of supernumerary teeth has been observed in 26-60% of patients with mean age around 9 years. Factors governed eruption of impacted incisors includes morphology, relative size, root formation, direction, space for eruption, and mesiodistal position of the supernumerary.

**Table 1:**
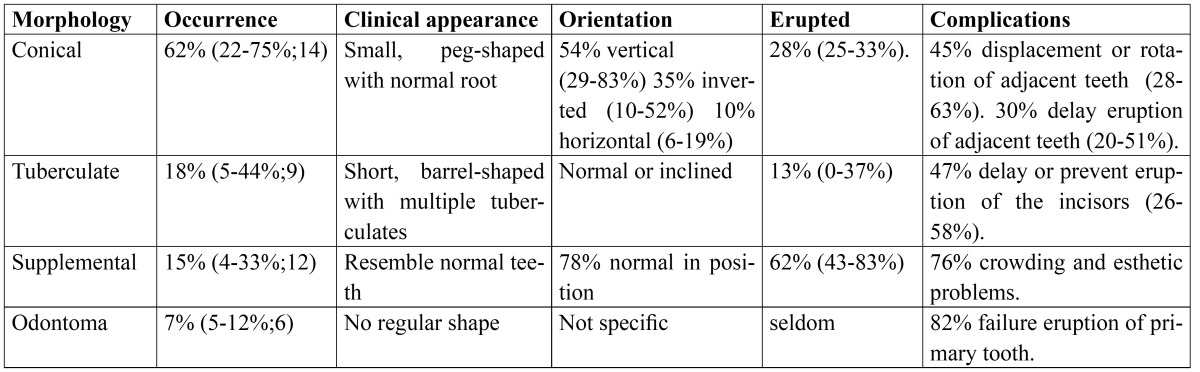
Frequency of supernumerary teeth in permanent dentition distributed according to morphology, location, and complications. Data in parenthesis represent the range of occurrence and number of studies.

Spontaneous eruption of impacted permanent incisors following removal of supernumerary ranged between 39% and 78% (4,8-10). Factors influence the rate of eruption include: type of supernumerary, availability of arch space, degree of displacement and inclination of impacted tooth, time of diagnosis and surgical intervention. In 35-50% of supernumeraries in primary dentition are superseded by extra teeth in the same location in permanent dentition.

- Etiology

The etiology of hyperdontia appears to be multifactorial; primarily polygenetic with some environmental influence. A high frequency of occurrence of multiple supernumerary teeth, ranged between 22 to 28 percent of the cases, are associated with certain developmental disorders such as cleft lip and palate, cleidocranial dysplasia and Gardener’s syndrome. Less frequent hyperdontia has been reported in Hallermann-Streiff syndrome; oral-facial-digital syndrome; Ellis-van Creveld syndrome; median cleft facial syndrome; Fabry-Anderson syndrome; Ito syndrome [incontinentia pigmenti achromians]; tricho-rhino-phalangeal syndrome, and Ehlers-Danlos syndrome (4,11,12). Multiple supernumeraries may also occur in non-syndrome cases (13).

- Prevalence

The prevalence of supernumerary teeth ranges from 0.03 to 1.9% in the primary dentition and 0.15 to 3.8% in permanent dentition (1-4) with the prevalence in the primary dentition about 5 times lowers. Beside racial variations, the age, size and type of the sample studied and the methodology used for detection, may account for this wide range. Males are affected approximately twice as frequently as female in the general Caucasian population. A greater male: female ratio was found among Mongolian groups (14).

- Classification 

Supernumerary teeth are classified according to morphology and location (3-6). In the primary dentition, supernumeraries are usually normal or conical in shape. In the permanent dentition, they have greater variety of forms. There are two morphologic types of teeth: supplemental [eumorphic or incisiform] rudimentary [or dysmorphic]. Rudimentary forms include conical, tuberculate, molariform, and odontoma types. Classification based on location includes mesiodens, distomolar, parapremolar, and paramolar.

- Clinical features and complications

A supernumerary tooth are frequently discovered when normal tooth is either delayed in its eruption or displaced. It may also be discovered by chance as a radiographic finding with no associated complications. Different types of supernumeraries have been associated with different effects on the adjacent teeth ( Table 1). Clinical complications caused by supernumerary tooth were observed in 46-88% of the patients. Supernumerary teeth, particularly in the permanent maxillary anterior region may cause the following clinical problems: delayed or failure of eruption of adjacent teeth incisors [26-60%]; displacement or rotation [28-63%]; crowding and malocclusion, retained primary teeth, root resorption of adjacent teeth, ectopic eruption, abnormal diastema [5-8%]; cystic formation [4-9%]; loss of vitality of the adjacent teeth [3.7%]; bone destruction, dilacerations; eruption into the nasal cavity; maxillary sinus or chin; pain and swelling at the site of involvement (3-5,9,14-16).

## Double Tooth

A double tooth is a rare developmental anomaly referring to fusion of two adjacent tooth buds or germination of single bud occurring during proliferation stage of tooth development. The clinical appearance is variable, ranging from a wide crown with a small notch of the incisal edge to almost two separate teeth. Double teeth are predominantly found in the anterior teeth of the maxilla or mandible in the primary or permanent dentition. In contrast to other dental anomalies, double teeth appear more frequent [about five times] in the primary than in permanent teeth (17,18). Fusion is most often seen in the mandibular primary incisors, while gemination usually occurs in the maxillary incisors and canines.

Fusion of teeth is the embryological union of two adjacent teeth germs, causing the formation of a single tooth with confluence dentin and enlarged clinical crown. Depending on the timing and type of interference during odotogenesis, fusion may be either completed or incomplete. Complete fusion occurs during the early stage of tooth formation that give rise to a single large crown with no apparent separation [groove] and a common pulp chamber. Incomplete fusion occurs in the late stage of odotogenesis may give a rise to nearly “separate” teeth or a single tooth almost twice the normal size. There is usually a facial groove extend from the incisal notch toward the cervical margin giving the crown a bifid appearance.

Gemination is an abortive attempt of a single tooth germ to divide by an invagination to form either 2 completely or incompletely separated crowns. The crown is large, demarcated by a shallow fissure or deep groove extend vertically from the incisal notch. The notch may advance to the middle of the crown.

- Etiology

Fusion and germination are opposite development conditions that involve alterations in tooth morphology. In fusion, the union of two tooth buds forms one tooth while in germination one tooth attempt to split in two. Fusion may occur due trauma during tooth development or crowding of adjacent tooth germs. On the other hand, gemination may result from the persistence of dental lamina between tooth germs. These developmental events appear to be influenced by hereditary and environmental factors. The unique occurrence of gemination, supernumerary tooth and talon cusp in the same tooth [presented in this case report] indicates that local hyperactivity of the dental lamina or its remnants may continue from the bud stage to morphodifferentiation. Double teeth may also be part of some systemic disorders such as chondroectodermal dysplasia [Ellis-van Creveld syndrome]; achondrodysplasia; focal dermal hypoplasia; otodental dysplasia; median cleft facial syndrome; oral-facial-digital syndrome and Russel-Silver syndrome.

- Prevalence

The prevalence of double primary teeth is 0.1 to 0.9% in white children and 1.55 to 3.0% in Asian children. In permanent teeth the frequency varied from 0.2 to 0.72%. No sex predilection has been found. The frequency of fused teeth ranges from 0.1 to 0.85% and gemination of 0.08 to 2.5%. The prevalence of triple teeth is 0.02%.

- Clinical features and complications

The clinical and radiographic appearance of double teeth is variable. To differentiate between fusion and gemination, dental history, clinical and radiological examinations are required. The “tooth counting rule” is used by counting the anomalous crown as one unit. A normal number of teeth per quadrant indicate gemination, whereas reduced number of teeth indicates fusion. An exception to this rule include: [1] fusion between supernumerary and normal tooth germs, [2] tooth congenitally missing, [3] fusion and gemination occur concurrently, [4] history of extraction or exfoliation. Radiographically, fused teeth may exhibit two pulp chambers, separated roots and root canals. In contrast, gemination usually present with a single enlarged pulp chamber, single root and root canal. However, differentiation between fusion and germination may become difficult when fusion involves a normal tooth and a supernumerary tooth. In primary dentition differentiation between fusion and germination is of limited clinical importance but it should draw clinician’s attention to the oncoming effects in the permanent dentition. It has been reported that double primary teeth have an influence on permanent successors up to 80% of cases (19,20). The most common problem related to fused primary teeth is hypodontia of the succedaneous teeth in 50 to 77% of cases, repeated double teeth [2.9%] and peg-shaped laterals [1.5-4%]. In rare clinical presentations, geminated teeth may associate with talon cusps or supernumerary teeth (21,22).

Clinical problems associated with double teeth:

1. Caries formation in the groove dividing the bifid crown.

2. Periodontal disease due to extension of the groove to the root surface.

3. Excess arch space and diastema occurs when normal teeth are fused.

4. Crowding of the dental arch, if the fusion involves one normal tooth and a supernumerary tooth.

5. Aplasia of the permanent successor in case of fusion.

6. Delayed exfoliation of primary double teeth.

7. Delayed root resorption and impaction of the permanent successor.

8. Malocclusion.

9. Esthetic problems.

## Talon Cusp

Talon cusp is a developmental dental anomaly referring to an accessory cusp-like structure projecting from the cingulum area or cementoenamel junction of the maxillary or mandibular anterior teeth in either the primary or permanent dentition (23). Mellor and Ripa (24) named the accessory cusp as talon cusp because of its resemblance in shape to an eagle’s talon. Because of differences in the form and location, the term dens evaginatus should be reserved for anomalous cone- or tubercles-shaped structure on the occlusal surfaces of posterior teeth. Talon cusp is composed of normal enamel and dentin and may or may not contain pulpal tissue. Palatal talon cusps represent the extreme of a continuous progressing from enlarged cingulum to a prominent well-defined accessory cusp extending to the level of the incisal edge forming with the incisal edge a T-shaped or, if lower in level, a Y-shaped crown contour. Some talon cusps are quite sharp and spike-like, while others have rounded and smooth tips. It can be horn-like, conical, pyramidal, bifid, or tubercle-like cingulum (25). The tip of the cusp may stand away from the rest of the crown or it may be in close approximation to the palatal surface of the crown. Talon cusps may be found on single or double tooth, unilateral or bilateral, and on the palatal / lingual or labial aspects of the affected [taloned] teeth.

- Review of literature

Since talon cusp was first described by Mitchell (26) in 1892, it was not until 1970 that talon cusp was again mentioned in case reports (24). A review of the literature up to1996 revealed 28 articles on talon cusps published in English language dental journals (25,27-29). Of these articles, 19 were on permanent teeth and described 63 cases with 89 taloned teeth. The nine reports of talon cusp in primary teeth involved 24 cases with 32 taloned teeth, in which seven cases had bilateral talon cusps (27). Males are more frequently affected than females but the ratio was considerably higher in primary teeth [3.5:1] than in permanent teeth [1.8:1]. The higher gender ratio of primary teeth could be related to the limited number of diagnosed primary taloned teeth compared with the affected permanent teeth [27 primary versus 94 permanent teeth]. Because of the increased awareness of the clinical problems associated with talon cusp, nearly 115 articles have been published during the past 15 year, representing 68% of the entire publication on this subject. They described 152 cases with 218 taloned teeth, including 172 [79%] in the permanent and 46 [21%] in the primary dentition.

To date, the total articles published on talon cusp are about 170 described 252 cases with 375 talon cusps on the palatal / lingual aspect and 32 cusps [25 cases] on the facial surface. Of the total 407 taloned teeth, 309 [76%] were permanent and 98 [24%] were primary teeth; with a ratio 3.1:1. Ninety-two percent of the cases were found in the maxilla and only 8% in the mandible. Among the permanent taloned teeth, 185 [60%] were central incisors, 105 [34%] lateral incisors and 19 [6%] canines. The affected teeth in primary dentition are distributed as follows: central incisors 73%, lateral incisors 21%, and 6% canines. For the facial talon, 65% was found on the central incisors, 22% on the lateral incisors and 13% on the canines. Bilateral talon cusp was recorded in 28 cases. The male to female ratio was 1.8:1 for both primary [37:20] and permanent [125:70] cases. In a review of primary taloned teeth, Lee et al. [2005] reported male to female ratio 1.8:1 [31:17 cases] (30).

- Etiology

The etiology of talon cusp is not completely understood. It may occur as a result of outward folding of inner enamel epithelial cells [precursors of ameloblasts] and a transient focal hyperplasia of the mesenchymal peripheral cells of dental papilla [precursors of odontoblasts] (23). Reports on bilateral occurrence of talon cusp, affected twins, siblings or parents, familial tendency, and racial differences suggest a significant genetic component in the etiology. It has been found that out of 13 affected cases 8 [62 %] had first-cousin parents (23,25). Whether family members are affected as a result of a shared genes or similar environmental condition is not yet elucidated. It appears that talon cusp has a multifactorial etiology combining both genetic and environmental factors. The talon cusp has not been reported as an integral part of any specific syndrome, although it appears more prevalent in patients with Rubinstein-Taybi syndrome, Mohr syndrome [oral-facial-digital II], Struge-Weber syndrome [encephalo-trigeminal angiomatosis], incontinentia pigmentia achromians, and Ellis-van Creveld syndrome [chondroectodermal dysplasia], hyopmelanosis of Ito, and Alagille’s syndrome (11,25,31).

- Prevalence

The prevalence of talon cusp varies considerably in different populations ranged from 0.06 to 7.7%. This anomaly occurs with a higher incidence in Mongoloid and Arab populations than in Caucasians and Negroes. In addition to the racial variations, the lack of precise criteria to classify an accessory cusp as “talon” has contributed to the extensive variations in prevalence. To standardized the terminology and diagnostic criteria, Hattab *et al*. (25) classified this anomaly into 3 types: Type 1 [true talon], Type 2 [semi talon], and Type 3 [trace talon]; based on degree of their formation and extension. Based on this classification, a survey on Turkish population showed that the most type in 44 taloned teeth was Type 1 (52.3%), followed by Type 2 [34.1%] and Type 3 [13.6%] (32). The survey showed that maxillary lateral incisors were the most affected teeth [66%] and canines were the least affected [9%]. The present review of literature showed that the maxillary central incisors were the most affected and canines were the least involved teeth; account only 6% [19:309] of taloned permanent teeth. A recent retrospective study on Jordanian sample, based on periapical radiographs, showed that maxillary canines were the most commonly affected teeth [46%; 23:52] (33). Apparently, the Jordanian study suffers from systematic errors in interpretation of radiographs.

- Clinical features and complications

Talon cusps vary in size, shape, structure, location, and clinical problems. Typical palatal / lingual or facial talon cusps extend from cementoenamel junction to the incisal edge. Radiographically, the talon cusp is visible as a V-shaped radiopaque structure superimposed on the image of the affected crown, with the point of the “V” towards the incisal edge. The cusp image is outlined by two distinct white lines, representing the enamel, converging from the cervical area towards the incisal edge. In true talon cusp pulpal extension could be traced to the cervical third of the anomalous cups.

Talon cusps are not an innocuous anomaly because large prominent cusps may present a number of clinical problems including traumatic occlusion; displacement of the affected and opposing teeth; compromised esthetics in cases of facial talon cusp; plaque retention and caries susceptibility in the developmental grooves delineate the cusp; periodontal problems; hypersensitivity, pulpal necrosis and periapical pathosis due to excessive attrition; attrition of the opposing teeth; accidental cusp fracture; irritation of tongue during speech and mastication; interference with tongue space; speech disturbance; breast-feeding problems; and temporomandibular joint pain due to excessive occlusal forces (26,28,30,31). A study of 57 taloned primary incisors showed that 18 of the 23 cases [78.3%] were associated with odontogenic abnormalities in which 14 cases associated with supernumerary teeth in the permanent successor (34). Developmental dental anomalies reported to be associated with talon cusp in-clude peg-shaped lateral incisors, ectopic or impacted canine, hypodontia, supernumerary tooth, double teeth, odontomes, dens evaginatus, dens invaginatus, shovel-shaped incisors, megadont and exaggerated Carabelli cusp (23-25,27,31,35). One-fourth of the reported taloned teeth had carious developmental grooves.

## Case Report

A 10 year old Jordanian-Arab girl presented to the dental clinic with complaint of large, unsightly upper front tooth. Her medical and dental histories were unremarkable and the patient had no record of orofacial trauma. No other members of the family are affected by similar dental abnormality. The child appeared healthy and of appropriate physical growth for her age. There were no abnormal extra-oral findings.

**Figure 1:**
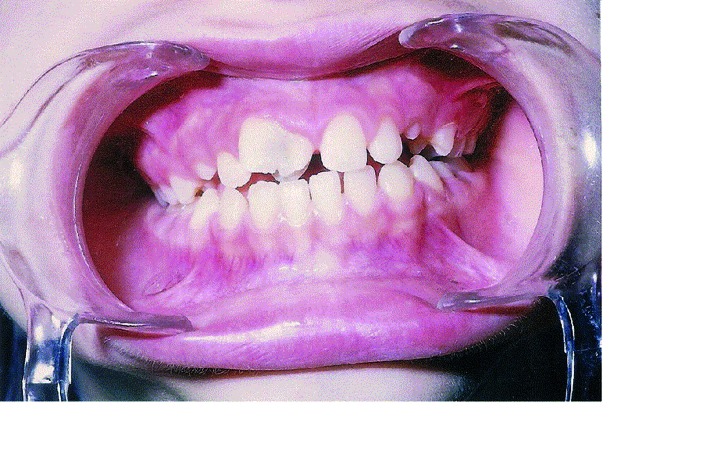
Frontal view showing right maxillary central fused to supernumerary tooth with premature contact of the talon cusps caused displacement and drifting of the affected tooth.

**Figure 2:**
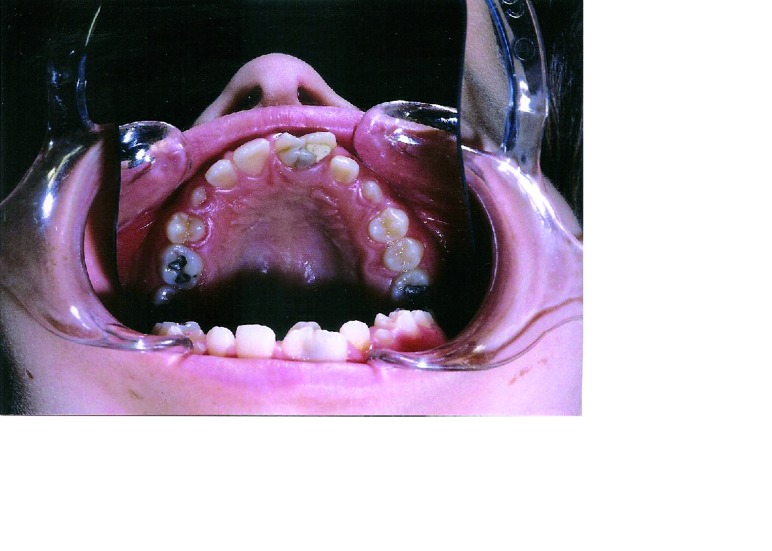
Mirror view of the palatal aspect showing two prominent talon cusps on the fused supernumerary tooth. There is a marked groove separating the cusps.

Intraoral examination revealed late mixed dentition with the left maxillary second molar was the only primary tooth retained. The permanent teeth were erupted at a normal chronological age. The occlusion was a class I molar relationship with palatally erupted maxillary right lateral incisor and midline shift 2 mm to the left in centric occlusion. Oral hygiene was fair and periodontal health good. The maxillary right central incisor was rotated and labially displaced. It had a large crown fused mesially to a supernumerary tooth with a marked labial groove at the site of fusion (Fig. 1). The supernumerary tooth had double joined talon cusps on the palatal aspect (Fig. 2). A deep groove separating the talon cusps was observed. The mesiodistal crown diameter of the anomalous tooth was 11.2 mm versus 7.3 mm of the contralateral central incisor, account 20% smaller in size than the counterpart in normal population (36). The anomalous tooth was not fully erupted. The accessory cusps were pronounced, well-defined extending from the cementoenamel junction to beyond the incisal edge [hyperocclusion]. Deep developmental grooves were present on the lateral aspect of the talon cusps at their junction to the palatal surface of the tooth. These grooves were packed with dental plaque and the distal groove was carious. A temporary filling was found on the depression of the affected central incisor. The affected tooth responded normally to electrical and thermal pulp tests. The cusps interferes with occlusion resulted in midline shift and wear-facet on the incisal edge of opposing tooth.

**Figure 3:**
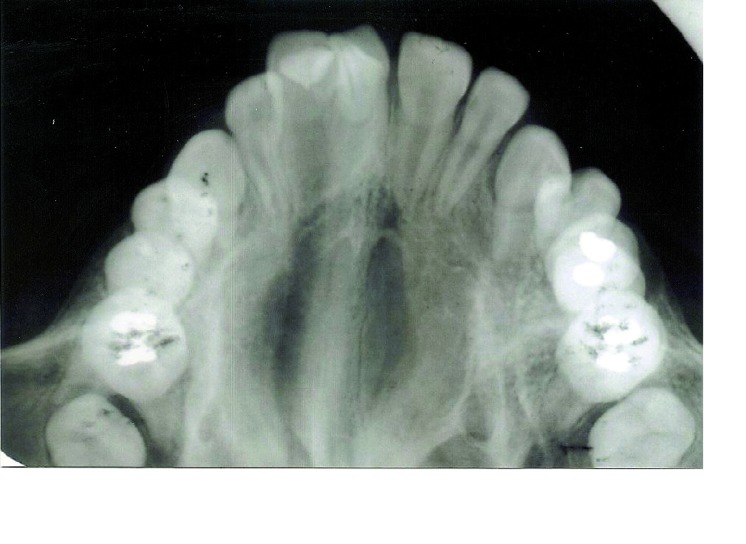
Occlusal radiograph depict central incisor fused along the entire length to the supernumerary tooth. Each tooth had a separate pulpal system and roots.

An occlusal radiograph (Fig. 3) showed the central incisor fused along the entire length to supernumerary tooth, with each tooth had a separate pulp chamber and root canal [incomplete fusion]. Based upon clinical and radiographic findings, a diagnosis of maxillary central incisor fused to doubletaloned supernumerary tooth was made. The fused supernumerary tooth exhibit V-shaped radiopaque structure superimposed on the image of the tooth, with the point of the “V” toward the incisal edge. The mesial talon cusp overlaps the distal one. Each cusp was outlined by two distinct white lines, representing the enamel, converging from the cervical area of the taloned tooth toward the incisal edge. Pulp extension could be traced radiographically to the tip of the cusp. A panoramic radiograph disclosed that all permanent teeth were present complete permanent dentition and developing at normal chronological age. Except the affected maxillary right central incisor, no other abnormalities were detected.

The treatment plan was based on the findings that the fused tooth had 2 separate roots and 2 separate pulp systems with a fusion groove separating the crowns. The plan was to hemisection the tooth along the fusion groove and extracts the conjoined supernumerary with its attached talon cusps. The treatment procedure and probable complication were explained to the parents. They showed no interest for surgical intervention but concerned about obtaining some improvement in the appearance and function. On the same visit, the part of talon cusp interfered with occlusion was removed using water-cooled diamond bur on a high-speed handpiece. The palatal carious groove was restored using flowable composite. Approximately 1.5 mm of the mesial and distal surfaces of the affected crown was reduced. The ground surfaces were treated with fluoride varnish [Duraphat®] as a desensitizing agent. The smaller crown size of contralateral incisor was increased using composite resin. Unfortunately, the patient failed to attend the clinic after the first visit.

## Discussion

- Supernumerary teeth

Early diagnosis of supernumeraries is crucial, if complications are to be avoided or minimized. Clinically the presence of supernumeraries should be suspected, if there is a prolonged delay in the eruption or displaced maxillary central incisors. Usually the problem is not noticed until the maxillary permanent lateral incisors start to erupt or have erupted and when one, or both, central incisors are missing and a primary predecessor(s) is present. Radiographic examination is essential to confirm diagnosis, the position of the supernumerary, its relation with the adjacent teeth, and the distance of the unerupted permanent teeth from the occlusal plane.

Management of supernumeraries depends on the age of the patient, type and position of the supernumerary, space available within the arch, and clinical problems created. Surgical removal of supernumerary in primary dentition is usually not recommended because of the risk of displacing and prejudices the vitality of the adjacent permanent teeth during the operation. Moreover, most primary supernumeraries erupt normally because of the presence of interdental spaces. Immediate removal of supernumerary tooth is indicated in the following cases: [1] adequate arch space is available, [2] significant delayed eruption or displacement of the adjacent teeth, [3] associated pathology, [4] interfere with orthodontic treatment, [5] erupted supernumerary.

The optimal time for surgical removal of an unerupted maxillary anterior supernumerary is controversial. Some authors advocate immediate removal of the supernumerary teeth following initial diagnosis of their presence, while others favour postponement of surgical intervention until the age of eight to ten years; when the root development of the incisors is nearly completed and thus reduce the risk of possible damage of the adjacent teeth during the operation (3,4,6,9)

Early removal of the offending supernumerary tooth, causing incisor impaction, may have the benefit of minimizing loss of eruptive potential, space loss, and midline shift. It also results in self-correction and proper alignment, if anterior arch space is available. The greatest concern with early removal is the risk of affecting the developing adjacent roots, resulting in loss of vitality and malformation. Additionally, a young child may be unable to tolerate the surgical procedure. A less conservative approach entails immediate surgical removal of the supernumerary and exposure of the unerupted tooth with or without orthodontic traction.

- Double teeth

Treatment of double teeth depends on clinical, radiographic evaluation and the requirements of the condition. This include the morphological variation of fused and geminated teeth, position, effect on adjacent teeth and successors, esthetic and periodontal problems, malocclusion and whether the affected teeth are primary or permanent. If the affected teeth are primary and not associated with functional problem or had no deleterious effects on the primary successors, they may retain in place until exfoliation. In case that double primary tooth retard development, delay or ectopic eruption of the successor, extraction and “pedo partial” is required. It is important to determine first whether the permanent successor is present.

Permanent double teeth present serious problems relating to esthetics and malocclusion. Multidiscipline treatment modalities have been suggested (37,38). Most of the fusion cases reported was fused incisor to supernumerary tooth. Treatment of fused tooth depends whether the tooth has a common pulp chamber and single root or 2 separate roots with no communication between the dental pulp of each tooth. In the first case, endodontic treatment may require, followed by reduction of the crown size [reshaping], reconstruction using composite resin or crown, and orthodontic treatment. Pronounced grooves at the union between fused teeth are susceptible to caries and should be sealed. In the second case, separation of fused teeth [hemisecting] is an option but does carry some risks. Complications to hemisecting include hypersensitivity, irreversible pulpitis, and external root resorption. Extraction of fused tooth is indicated in cases of unfavourable endodontic treatment or impeding the eruption of adjacent tooth. The removed tooth is replaced with an interim removable partial denture until fixed bridge or implant is constructed. In cases of gemination where there are esthetic concerns and no underlying risk factors; crown reshaping, direct composite bonding, and orthodontic treatment have been described (21).

- Talon cusps

The treatment of talon cusps requires careful clinical and radiographic judgment because some types of talon cusps caused a number of clinical problems. On unerupted tooth, the anomalous cusp can radiographically be mistaken for a supernumerary tooth or compound odontomas, leading to unnecessary surgical intervention. This diagnostic problem is especially significant because the majority of talon cusps and supernumeraries occur in the maxillary central and lateral incisor region. The treatment objectives for taloned teeth include preserving tooth vitality, esthetic restoration, elimination of occlusal interference, eradication of carious developmental grooves, and sealant of susceptible grooves. Small cusps are usually asymptomatic and need no treatment. Large prominent, sharply defined cusp associated with functional, pathological, esthetic and orthodontic problems call for early and definitive treatment.

Reports, based on radiographic examination, indicated that talon cusp contain pulp horn extension. However, radiographic tracing of the pulpal configuration inside the talon cusp has inherent difficulties because the cusp and its pulpal extension are superimposed over the affected crown. Histological examination of extracted or exfoliated taloned teeth either failed to reveal a pulpal extension into the cusp or confirmed the presence of pulpal tissue. Hattab *et al*. (23,25) removed 1 to 1.5 mm from several talon cusps in one visit or complete removal of the cusp on 2 consecutive appointments of 6 to 8 week interval (21); without exposing the pulp. After each reduction, the ground surface was treated with fluoride varnish [Duraphat®] as desensitizing agent and to enhance reparative dentin formation. Stanley *et al*. (39) reported that the daily rate of reparative dentine formation after operative procedure in permanent teeth is 1.49 µm. It seems that large talon cusps projecting away from the tooth crown are more likely to contain pulp tissue. Radical removal of such cusps may result in pulp exposure. Treatment option for such cases includes pulpotomy, partial pulpectomy, root canal therapy or extraction followed by orthodontic treatment.
